# Decoding effects of psychoactive drugs in a high-dimensional space of eye movements in monkeys

**DOI:** 10.1093/nsr/nwad255

**Published:** 2023-10-03

**Authors:** Xu Liu, Zhixian Cheng, He Lin, Jiangxiu Tan, Wenyao Chen, Yichuan Bao, Ying Liu, Lei Zhong, Yitian Yao, Liping Wang, Jijun Wang, Yong Gu

**Affiliations:** Shanghai Key Laboratory of Psychotic Disorders, Shanghai Mental Health Center, Shanghai Jiao Tong University School of Medicine, Shanghai 200030, China; Lingang Laboratory, Shanghai 200031, China; The Third Research Institute of Ministry of Public Security, Shanghai 200031, China; CAS Center for Excellence in Brain Science and Intelligence Technology, Institute of Neuroscience, Chinese Academy of Sciences, Shanghai 200031, China; CAS Center for Excellence in Brain Science and Intelligence Technology, Institute of Neuroscience, Chinese Academy of Sciences, Shanghai 200031, China; CAS Center for Excellence in Brain Science and Intelligence Technology, Institute of Neuroscience, Chinese Academy of Sciences, Shanghai 200031, China; CAS Center for Excellence in Brain Science and Intelligence Technology, Institute of Neuroscience, Chinese Academy of Sciences, Shanghai 200031, China; CAS Center for Excellence in Brain Science and Intelligence Technology, Institute of Neuroscience, Chinese Academy of Sciences, Shanghai 200031, China; CAS Center for Excellence in Brain Science and Intelligence Technology, Institute of Neuroscience, Chinese Academy of Sciences, Shanghai 200031, China; CAS Center for Excellence in Brain Science and Intelligence Technology, Institute of Neuroscience, Chinese Academy of Sciences, Shanghai 200031, China; University of Chinese Academy of Sciences, Beijing 100049, China; Shanghai Key Laboratory of Psychotic Disorders, Shanghai Mental Health Center, Shanghai Jiao Tong University School of Medicine, Shanghai 200030, China; Institute of Psychology and Behavioral Science, Shanghai Jiao Tong University, Shanghai 200030, China; CAS Center for Excellence in Brain Science and Intelligence Technology, Institute of Neuroscience, Chinese Academy of Sciences, Shanghai 200031, China; University of Chinese Academy of Sciences, Beijing 100049, China

**Keywords:** oculomotor, monkey, schizophrenia, phencyclidine, ketamine, auditory evoked potential

## Abstract

Oculomotor behavior has been shown to be correlated with mental disorders in clinics, making it promising for disease diagnosis. Here we developed a thorough oculomotor test toolkit, involving saccade, smooth pursuit, and fixation, allowing the examination of multiple oculomotor parameters in monkey models induced by psychoactive drugs. Eye movements were recorded after daily injections of phencyclidine (PCP) (3.0 mg/kg), ketamine (0.8 mg/kg) or controlled saline in two macaque monkeys. Both drugs led to robust reduction in accuracy and increment in reaction time during high cognitive-demanding tasks. Saccades, smooth pursuit, and fixation stability were also significantly impaired. During fixation, the involuntary microsaccades exhibited increased amplitudes and were biased toward the lower visual field. Pupillary response was reduced during cognitive tasks. Both drugs also increased sensitivity to auditory cues as reflected in auditory evoked potentials (AEPs). Thus, our animal model induced by psychoactive drugs produced largely similar abnormalities to that in patients with schizophrenia. Importantly, a classifier based on dimension reduction and machine learning could reliably identify altered states induced by different drugs (PCP, ketamine and saline, accuracy = 93%). The high performance of the classifier was reserved even when data from one monkey were used for training and testing the other subject (averaged classification accuracy = 90%). Thus, despite heterogeneity in baseline oculomotor behavior between the two monkeys, our model allows data transferability across individuals, which could be beneficial for future evaluation of pharmaceutical or physical therapy validity.

## INTRODUCTION

As non-competitive N-methyl-D-aspartate (NMDA) receptor antagonists, phencyclidine (PCP) and its derivative ketamine have the potential to disrupt glutamatergic neurotransmission [1,2], thereby contributing to their unique clinical and psychoactive effects. PCP was used as an anesthetic in the 1950s, but its clinical application was soon restricted due to emerging adverse side-effects including hallucinations, delusions, anxiety, cognitive deficits, and other schizophrenia-like symptoms [2]. Recently, PCP has gained renewed attention for cognitive research and pharmacological models of schizophrenia [3]. Analogously, ketamine is also an anesthetic, with side-effects similar to PCP-induced schizophrenia-like symptoms [4]. These symptoms however, are relatively milder and shorter-lasting [5], thus ketamine is more widely used in clinics, such as anesthesia and anti-depressive therapy [6]. In addition, ketamine has also been widely used in cognitive research experiments on drug-induced animal models [4]. To date, most of the animal models induced by PCP and ketamine, such as rodent and primate models, focus on deficits in cognitive functions including working memory, cognitive control, attention etc. [2,7–10]. While these measurements provide valuable insight into underlying neurobiological mechanisms of schizophrenia-like cognitive disorders, and help develop new medicine treatment or physical intervention, it remains a challenge for validity and reliability of schizophrenia-like cognitive deficit in animal models.

On the other hand, the oculomotor system, particularly in visually guided animals such as primates, involves a complex neural network including cortical, subcortical brainstem and cerebellar areas, responsible for controlling and coordinating various types of eye movements including rapid saccade, smooth pursuit, fixational eye movements, and pupillary response [11,12]. These plentiful oculomotor behaviors not only mediate simple reflex-like eye movements for orienting attention or maintaining fixation stability, but also reflect more complicated cognitive control, or flexible decision-making in complex environments when confronting multiple sources of contradicting information. Thus, oculomotor behaviors are sensitive to deficits within the underlying neural network, and may show more robust effects when compared to those complex cognitive tasks that typically suffer from too much variability across individuals. Hence, oculomotor tests are potentially useful for clinical usage due to their reliability and convenience. Indeed, aided with state-of-the-art noninvasive eye trackers with ultrahigh temporal and spatial resolutions, oculomotor tests have recently become a popular tool for examining mental disorders such as schizophrenia [13,14], depression [15], attention deficit hyperactivity disorder (ADHD) [16], Parkinson disease (PD) [17], and Alzheimer disease (AD) [18].

Patients with schizophrenia, for example, have been shown to exhibit characteristic oculomotor behaviors in numerous studies. Briefly, they have difficulty in inhibiting reflexive saccade and in initiating correct saccade in cognitive-control tasks such as anti-saccade and delayed saccade [19,20]. For smooth pursuit, they show deficits in the pursuit quality, reflected by making more saccades and lower pursuit gain [21]. During fixation, the patients typically exhibit less stable fixation [22], abnormal microsaccades [14], and reduced pupillary response [23]. Taken together, these abnormal oculomotor behaviors are closely related to cognitive deficits and other psychotic symptoms, reflecting underlying disruptions in neural circuitries. Therefore, oculomotor behaviors may be promising biomarkers for early diagnosis, as well as for treatment, of mental disorders.

In the current study, we aim to develop an oculomotor test toolkit in nonhuman primates, specifically, macaques. Compared to other popular animal models such as mouse, the macaque exhibits a much higher degree of similarity in brain structure and function with humans. Thus, macaques are more likely to develop similar mental disorders, for example, schizophrenia-like symptoms as seen in humans. Importantly, both humans and macaques rely highly on visual information in their daily life, and they possess nearly identical brain circuits for complex eye movement control, including both saccade and pursuit systems. Thus, developing an oculomotor test toolkit on macaque models would greatly help to further investigate underlying neural mechanisms, as well as test intervention techniques including physical modulation and medicine treatment. Thereby, we developed a toolkit that can particularly test many oculomotor behaviors including saccade, pursuit, and fixation, allowing us to build up a high dimension space of eye movement parameters on the same individual subject. We then tested this model by applying PCP and ketamine, and examined how different states (i.e. saline-, PCP-, or ketamine-application) could be decoded from this high-dimensional space.

## RESULTS

Two macaques were first trained to perform a test toolkit of oculomotor behavior that mainly fell into three categories: saccade, smooth pursuit, and fixation (Fig. S1A). In particular, the saccade task contained two tests: anti-saccade and delayed saccade. The pursuit task also contained two tests, including a two-dimensional Lissajous smooth pursuit, and a one-dimensional linear pursuit. The fixation test contained simple fixation of a visual spot at the center of the visual display. In addition to these instructed tasks, the animals also experienced free-viewing conditions in which there were no instructed eye movements.

After the animals reached stable performance, we conducted acute drug-injection experiments with PCP (0.3 mg/kg) [9] and ketamine (0.8 mg/kg) [8] delivered from muscles in lower limbs. Oculomotor performance was collected after drug injection (see Methods and Fig. S1). The PCP and ketamine tests were conducted separately. In total, monkey Y received 106 injections in PCP tests (48 PCP and 58 saline) and monkey N received 111 injections (50 PCP and 61 saline). In the ketamine tests, both monkeys received 58 injections (24 ketamine and 34 saline) (Fig. S1B). Oculomotor data in each task were analyzed by focusing on several parameters including: (1) accuracy, reaction time, saccade velocity, saccade duration and saccade amplitude in saccadic tasks: (2) pursuit gain and saccade occurrence rate in pursuit tasks; (3) microsaccadic performance, fixation ability and pupillary response in the fixation test. In parallel, auditory evoked potential (AEP) in the electroencephalogram in response to repeated auditory cues were also collected and compared before and after drug injection. In the following, we illustrate the impact of drug injections on these parameters, respectively.

### Effects on anti-saccadic performance

**Figure 1.**
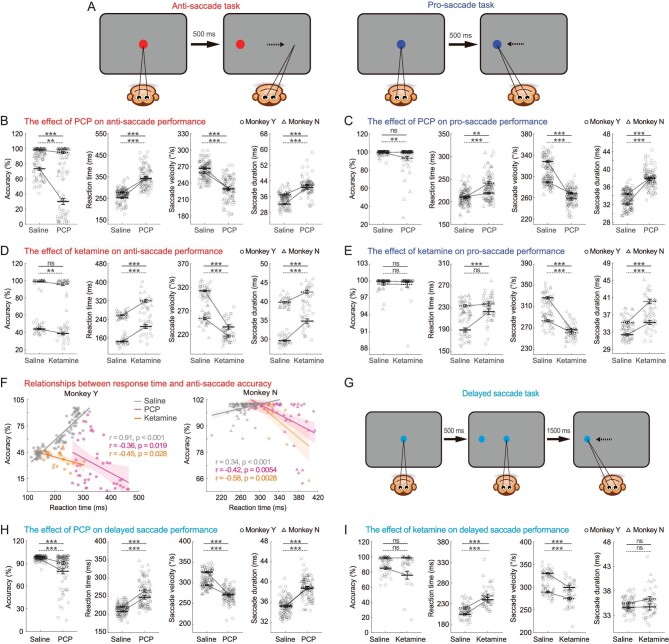
Effects of PCP and ketamine on saccadic performance. (A) Paradigm of anti- and pro-saccade task. Red cue indicated that saccade should be in the anti-direction of the peripheral target (left panel), whereas blue cue indicated saccade in the same direction (right panel). (B and C) PCP effects on task accuracy, reaction time, saccade velocity and saccade duration in anti- (B) and pro-saccade (C) trials. (D and E) Same as in B and C, with ketamine effects. (F) Relationships between accuracy and reaction time in anti-saccade trials. Grey symbols: saline control. Color symbols: PCP and ketamine injections. (G) Paradigm of delayed saccade task. Monkeys discarded the peripheral target by maintaining central fixation, until the fixation spot disappeared indicating a go-signal and then conducted a pro-saccade to the peripheral target. (H and I) Effects of PCP (H) and ketamine (I) on accuracy, reaction time, saccade velocity and saccade duration during the delayed saccade task. Circle and triangle represent data from two monkeys. Solid, dark symbol represent mean value and the open, light symbols are data from each session. Error bars indicate ± SEM. Statistical significance: *0.01 < *P* < 0.05; **0.001 < *P* < 0.01; ***<0.001; ns, *P* > 0.05 (solid line for monkey Y; dotted line for monkey N).

In the anti-saccade task, the animals confronted two types of instructions on their saccadic directions (Fig. 1A). In the anti-saccade trial, when the central-fixation spot and the peripheral-target were cued as red, the correct saccadic direction was in the opposite direction of the target's location (Fig. 1A, left panel). In the pro-saccade trial, the central-fixation spot and the peripheral-target were cued as blue, and the correct saccadic direction was in the same direction as the target's location (Fig. 1A, right panel). Anti-saccade and pro-saccade trials were interleaved within one experimental session (See Methods).

We first examined the effects of drug injection on the accuracy of saccadic direction and reaction time of the saccade initiation in anti-saccade trials. These two parameters are tightly related with the decision-making process in cognitive control tasks [24]. Compared to saline control, both PCP and ketamine injections significantly lowered the accuracy of saccade direction, and elongated the reaction time of saccade initiation (Fig. 1B and D, columns 1 and 2). Interestingly, both drugs reversed the relationship between the two decision-making parameters. Specifically, before injection, correct rate was positively correlated with reaction time, reflecting a typical speed-accuracy tradeoff strategy in making decisions (Fig. 1F, grey symbols), whereas after injection, the two parameters became negatively correlated with each other (Fig. 1F, color symbols).

We then examined the drug effects on the motor aspect of saccade by investigating its averaged velocity, duration, and amplitude for each eye movement. Both PCP and ketamine significantly decreased the saccadic velocity (Fig. 1B and D, 3rd column), and prolonged saccadic duration (Fig. 1B and D, 4th column). Yet the effects on the amplitude was heterogeneous, with PCP tending to increase while ketamine tended to decrease the parameter (Fig. S2A).

While oculomotor behavior in anti-saccade trials are mainly cognitive when confronting conflicting information (i.e. saccade to the opposite direction of the cued target), saccades in the pro-saccade trials are more intuitive because saccadic direction is consistent with the cued target (Fig. 1A, right panel). Overall, drug-injection affected pro-saccade behavior similar to that in the anti-saccade trials, albeit with some differences. In particular, overall accuracy was not significantly affected by PCP or ketamine in pro-saccade trials. The largely unaffected accuracy in the pro-saccade trials may be due to a ceiling effect of performance compared to that in the sensory-instruction conflict anti-saccade trials (Fig. 1C and E, 1st column). However, initiation of pro-saccade was still largely delayed (Fig. 1C and E, 2nd column). The averaged pro-saccade velocity was significantly reduced (Fig. 1C and E, 3rd column), and the pro-saccade duration was significantly prolonged (Fig. 1C and E, 4th column). The drug effect on the saccade amplitude was also significant, albeit with some heterogeneity (Fig. S2B).

### Effects on delayed saccade performance

Delayed saccade is another classical cognitive demanding task. The key is that subjects need to maintain central fixation by suppressing their instinctive saccade to an emerging peripheral target that could distract attention. Subjects can only make a saccade to the peripheral target when the central fixation spot disappears, indicating a ‘go’ signal (Fig. 1G).

Drug injections tended to impair the animals’ inhibition of reflexive saccade to peripheral targets. PCP induced significantly more breaks during the inhibition period (Fig. 1H, 1st column). Ketamine also produced a similar effect albeit with a smaller effective size and lack of statistical significance (Fig. 1I, 1st column). After the go-signal appeared, both drug injections elongated initiation of saccade (Fig. 1H and I, 2nd column). For the saccade action, both drugs reduced the average velocity (Fig. 1H and I, 3rd column). Moreover, PCP but not ketamine prolonged the saccade duration (Fig. 1H and I, 4th column). The saccade amplitude was also significantly affected, yet the effects were heterogeneous across subjects and drugs (Fig. S2C).

### Effects on smooth pursuit eye movements

Smooth pursuit is another common oculomotor behavior during daily life for maintaining visual stability of the intended target that is moving continuously in the environment. To examine drug-injection effects on smooth pursuit eye movements, we conducted two paradigms: a simple linear, one-dimension smooth pursuit with constant speed, and a more complicated, two-dimension Lissajous pursuit with varied speed in both dimensions.

**Figure 2.**
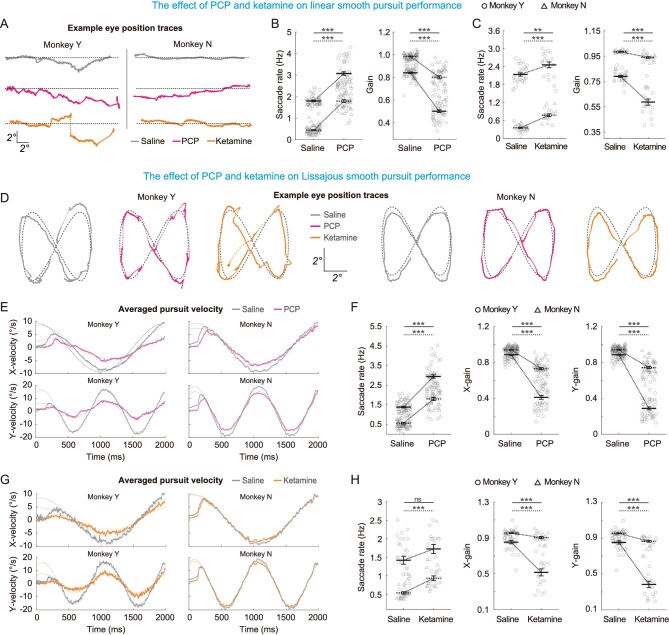
PCP and ketamine impaired smooth pursuit performance. (A) Linear smooth pursuit task with constant speed in one dimension and examples of eye position traces before (saline control) and after PCP (magenta) and ketamine (orange) injections. (B and C) PCP (B) and ketamine (C) induced more saccades and lower pursuit gain. (D) Lissajous pursuit that involved two-dimensional pursuit with varied speed. Example of eye position traces were in same format as in (A). (E) Averaged pursuit velocities (solid lines) in each cardinal axis of monkeys treated with PCP(s) and saline during pursuit tasks. Dotted lines represent fitting data. (F) PCP induced more saccades and lower x- and y-pursuit gains. (G and H) same as (E and F) but for saline and ketamine. Circle and triangle represent data from two monkeys. Solid, dark symbol represent mean value and the open, light symbols are data from each session. Error bars indicate ± SEM. Statistical significance: **0.001 < *P* < 0.01; ***<0.001; ns, *P* > 0.05 (solid line for monkey Y; dotted line for monkey N).

Figure 2A showed examples of eye trace when the monkeys tracked a visual target moving along one of the cardinal axes with a constant speed of 10 degrees per second. Both PCP and ketamine injections appeared to impair the pursuit quality. For example, both animals making more catch-up saccades, potentially due to the fact that their pursuit eyes could not keep up with the moving targets. To quantify this, we calculated the saccade frequency and pursuit gain (see Methods) during pursuit. We found that both PCP and ketamine injections significantly increased saccade frequency, and reduced pursuit gain (Fig. 2B and C).

Compared with the linear pursuit, the Lissajous pursuit task is more demanding because the target is moving on a plane, and its speed varies dynamically along the two cardinal axes (Fig. 2D). Thus, we analyzed data separately for the x- and y-axis (Fig. 2E and G). After drug injection, both animals showed difficulty in tracking the moving target, particularly when the target's motion reached a high speed (Fig. 2E and G). Again, both drugs induced more saccades during pursuit (Fig. 2F and H, 1st column), and dramatically reduced pursuit gain in both horizontal and vertical axes (Fig. 2F and H, columns 2 and 3).

### Effects on fixational eye movements

**Figure 3.**
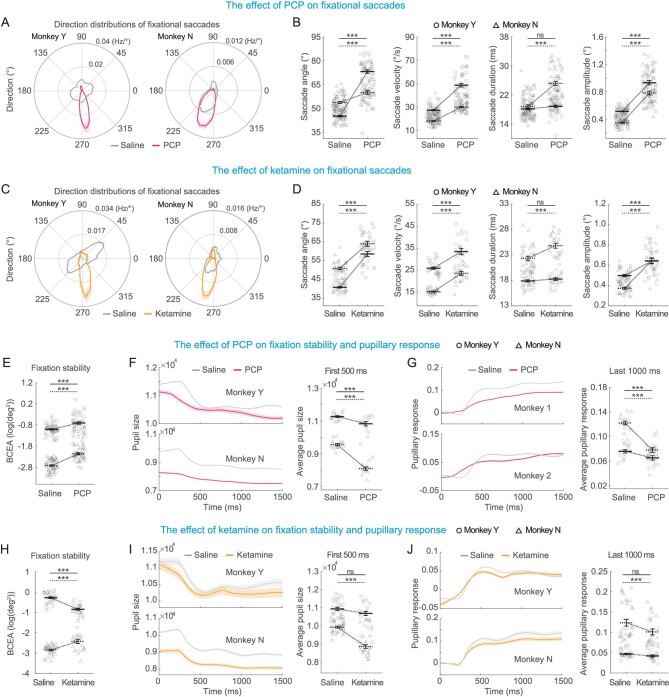
Effects of PCP and ketamine on microsaccades, fixation stability and pupillary response. (A) The averaged direction distributions of fixational saccades for monkeys treated with saline (gray) and PCP (magenta). (B) Summary result for microsaccade angles, defined as the deviation from the horizontal meridians (1st column), averaged velocity (2nd column), duration (3rd column) and amplitude (4th column). (C and D) same as (A and B) but for saline (gray) and ketamine (orange). (E) The bivariate contour ellipse area (BCEA) of monkeys treated with saline and PCP. The larger BCEA indicated the more instable fixation. (F) Time courses of pupil sizes (left) and average pupil sizes of first 500 ms (right) for monkeys treated with saline and PCP during visual fixation. (G) Time courses of pupillary responses (left), pupil-size change relative to baseline (average pupil size of first 200 ms), and average pupillary responses of last 1000 ms (right) for monkeys treated with saline and PCP. (H–J) same as (E–G) but for saline and ketamine. Circle and triangle represent data from two monkeys. Solid, dark symbol represent mean value and the open, light symbols are data from each session. Error bars indicate ± SEM. Statistical significance: ***<0.001; ns, *P* > 0.05 (solid line for monkey Y; dotted line for monkey N).

In addition to saccade and pursuit eye movements, we also examined oculomotor behavior during the central fixation period including three aspects: microsaccades, fixation stability, and pupillary response (Fig. 3).

Microsaccades usually happen involuntarily during the visual fixation period. They are very small saccades with amplitudes usually less than 2 degrees [25]. Before drug injection, microsaccades were distributed roughly uniformly in all directions in the fronto-parallel plane (Fig. 3A and C, gray lines). Yet after drug injection, they were significantly biased toward downward either by PCP or by ketamine (Fig. 3A and C, color lines). To quantify this, we computed a simple saccade angle index as the difference of the saccade direction and the horizontal meridian. It was clear that after drug injection, the indices were clustered toward vertical (Fig. 3B and D, 1st column). Interestingly, this directional bias is consistent with a recent study showing a greater vertical shift of microsaccades in patients with schizophrenia [14]. Finally, contrary to regular saccade, injection of the two drugs significantly increased microsaccadic velocity (Fig. 3B and D, 2nd column) and amplitude (Fig. 3B and D, 4th column) in both monkeys. Microsaccadic duration was also elongated particularly in one monkey (Fig. 3B and D, 3rd column). These results are consistent with recent findings in schizophrenia patients [14].

Fixation stability has also been shown to be impaired in schizophrenia patients [22]. We found that injection of the two drugs also tended to impair the monkeys’ fixation stability quantified by the bivariate contour ellipse area (BCEA) of the overall eye positions during central fixation. In particular, PCP consistently evoked a larger BCEA, indicating the fixation became less stable (Fig. 3E). Ketamine also induced a significant effect yet the effect was heterogeneous across monkeys: a larger BCEA in one monkey and a smaller BCEA in the other monkey (Fig. 3H), which might be due to the inhibition of horizontal eye movements (Fig. 3C left).

Pupil size and its changes were also measured during central fixation. Compared to saline control, both drugs tended to reduce the overall pupil size throughout the fixation period (Fig. 3F and I). In addition to the average pupil size, the change in pupil size within each trial, reflecting the response range, was also significantly reduced by PCP injection (Fig. 3G). Ketamine produced a similar effect yet it did not reach statistical significance in one monkey (Fig. 3J).

### Effects on free-viewing performance

In all aforementioned tasks, the animals were instructed to make specific eye movements. In this section, we removed the mandatory instructions, by presenting an image to the animals, and allowed them to view freely at the visual display. Reward was delivered randomly during this process without any associations to the behavior. Motivated by the theory that both hallucinations and visual illusions may arise from a similar predictive coding mechanism [26], we adopted classical illusory visual images that can induce strong motion illusions on human beings (see Supplementary Fig. S3). Since macaques and humans share many analogous visual information processing mechanisms, we believe that the animals should also observe these illusions, as has been proven in some experiments [27,28]. As a control, non-illusory images were also included, to help examine whether psychoactive drugs would induce any distinguished effects on the illusory and non-illusory images. The other advantage of these images is neutral without significant emotional information as may exist in the natural or face images that have been used in other studies. However, despite this effort, we found basically similar results across the two types of stimuli (Supplementary Fig. S3), consistent with previous findings that the effect of ketamine does not depend on the choice of images [29]. Thus, data from the two categories of images were pooled together for the following description.

**Figure 4.**
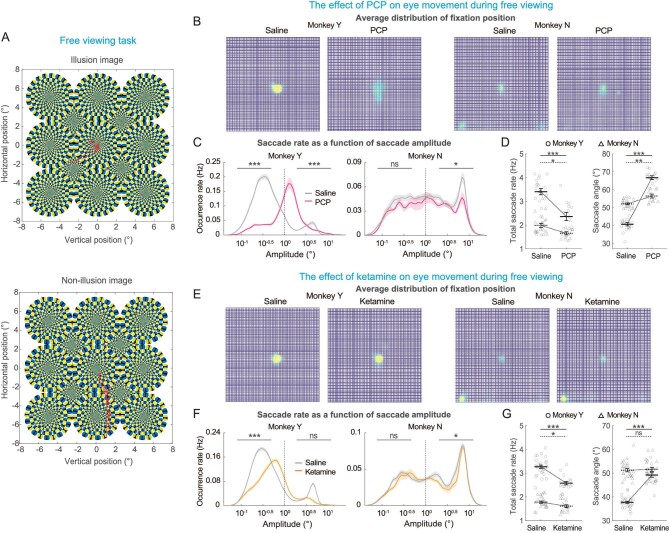
Eye movements during free-viewing tasks. (A) An illusory image (top) and non-illusory image (bottom) used, with exemplary eye movement (red line) superimposed on the images. (B) Distribution of eye position before and after PCP injection. (C) Proportion of saccade amplitude before (grey curves) and after PCP injection (color curves). (D) The overall saccade rate and direction away from horizontal meridian (saccade angle) before and after PCP injection. (E–G) same as (B–D) but for saline and ketamine results. Circle and triangle represent data from two monkeys. Solid, dark symbol represent mean value and the open, light symbols are data from each session. Error bars indicate ± SEM. Statistical significance: *0.01 < *P* < 0.05; **0.001 < *P* < 0.01; ***<0.001; ns, *P* > 0.05 (solid line for monkey Y; dotted line for monkey N).

First, we found that in the free-viewing context, the two monkeys appeared to show some different habit of gaze before drug injections. In particular, monkey Y mainly focused around the center of the images (Fig. 4B and E, 1st column), while monkey N focused around a few places including the center, lower left and lower right corner of images (Fig. 4B and E, 3rd column). Despite this difference of gaze location in the two animals, neither PCP nor ketamine injection dramatically changed individual's gaze habit (Fig. 4B and E, 2nd and 4th columns).

During free-viewing, the animals also frequently made saccades with different amplitude. For monkey Y, majority of saccades were as small as within 1°, indicating most likely they were microsaccades (Fig. 4C, left panel). PCP injection tended to increase the saccadic amplitude, consistent with the finding described previously during instructional tasks. For monkey N, saccade amplitude was more diversely distributed, and there was a peak around 6° (Fig. 4C, right panel), suggesting that this animal made larger saccade than the other animal. After PCP injection, these large saccades were slightly reduced. Overall, in both animals, PCP tended to reduce saccade frequency (Fig. 4D, left panel), and biased their direction toward downward in the vertical plane (Fig. 4D, right panel). These results for PCP were similar when applied to ketamine (Fig. 4F and G).

### Classification of drug-injection state

**Figure 5.**
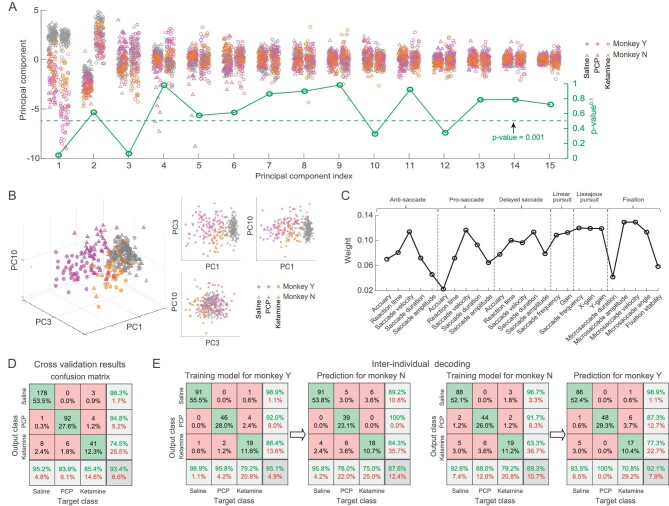
PCA and classification. (A) Scatterplot of principal components (PCs) (up to 15th PC) and statistical significance of PCs among three drug-injection states with one-way ANOVA (green). (B) The 1st, 3rd, and 10th PCs exhibiting the smallest *p*-values were plotted in a three-dimensional space (left), and plotted against each other (right), with gray, magenta and orange symbols indicating saline-, PCP- and ketamine-injections, respectively. Circle and triangle represent data from two monkeys. (C) The weights of oculomotor parameters loaded onto the 1st, 3rd, and 10th PCs. (D) Cross-validation results for the multi-class support vector machine (SVM) classifier based on the data mixed from the two monkeys. (E) Inter-individual decoding results.

Finally, we combined the oculomotor data measured from two monkeys, and used principal component analysis (PCA) and the multi-class classification method of Gaussian support vector machine (SVM) to classify the three drug-injection states: saline, PCP, and ketamine. After PCA, one-way analysis of variance (ANOVA) was used to analyze the statistical differences among the PCs in three injection states. The 1st, 3rd, and 10th PCs exhibited the smallest *p*-values (Fig. 5A, green line) and selected as the inputs for machine learning. These three components in a three-dimensional space (Fig. 5B, left panel), showed that the three injection states were largely separated from each other (PC1 vs. PC3, PC1 vs. PC10, Fig. 5B, right panel). Among all PCs, the PC2 largely captured variance in the two individual animals (Fig. 5A). We then calculated the weight of oculomotor parameters projected onto the three PCs (see Methods). The parameters with the top 10 weights are as follows: microsaccade velocity and amplitude during fixation, saccade frequency, horizontal and vertical gains in the Lissajous pursuit task, saccade velocities in pro-, anti-, and delayed saccade tasks, microsaccade angle, and pursuit gain in the linear pursuit task (Fig. 5C), indicating that these oculomotor parameters characterized mainly the three PCs.

Despite the fact that there were large individual differences between the two monkeys, the multi-class SVM decoder with Leave-one-out cross-validation algorithm could reach an accuracy rate of 93.4% in classifying among three injection states based on the data mixed from the two animals (Fig. 5D). Specifically, the accuracy rate for distinguishing between the drug-injection (PCP and ketamine) states and the saline control state reached 96.3%, and for distinguishing between the PCP- and ketamine-injection states reached 93%. These results suggest that the oculomotor behavior measurements could effectively be used to distinguish the three injection states based on oculomotor data mixed across subjects.

Furthermore, to test the transferability of the model, we performed analysis by training data from one monkey and testing drug-injection state in the other monkey. Specifically, we found that using data from monkey N to predict the state of monkey Y could achieve an accuracy of 92%. Reversely, using data from monkey Y to predict the state of monkey N could achieve an accuracy of 88% (Fig. 5E). These results imply potential for transferability of learned parameters from one individual another.

### Auditory evoked potentials (AEPs) were enhanced by drug injection

**Figure 6.**
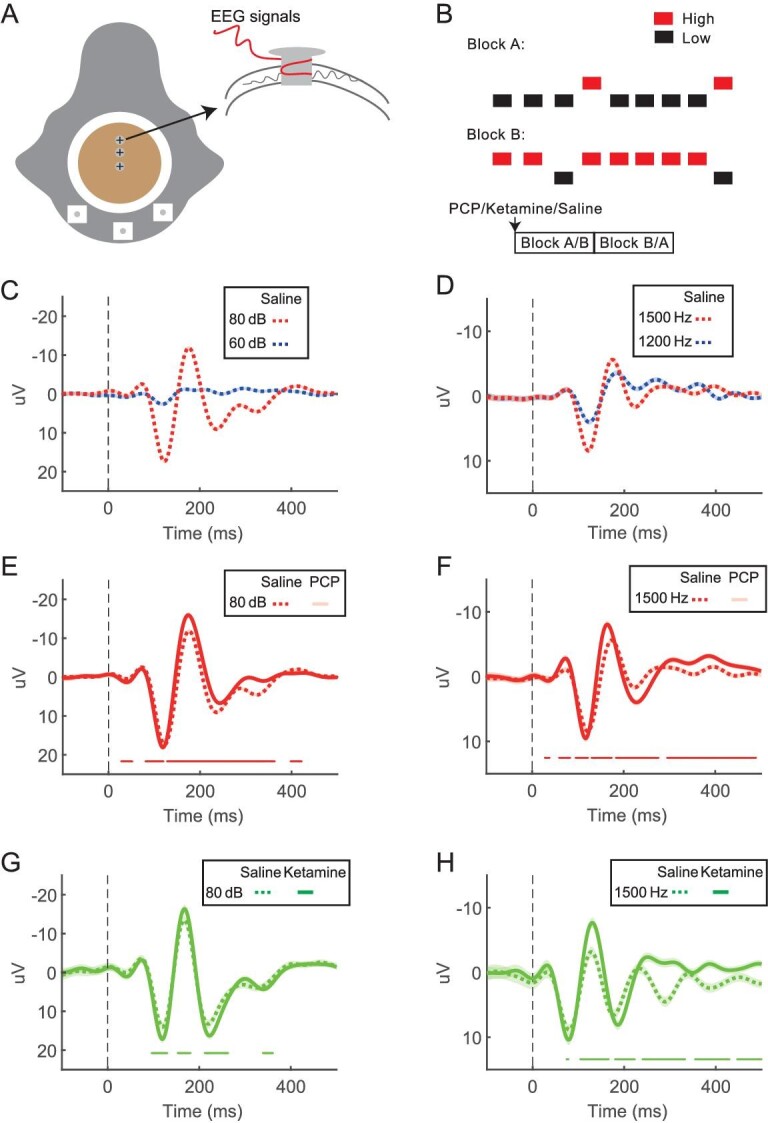
Auditory evoked potentials (AEP) were enhanced during PCP/ketamine sessions. (A) The configuration of electrodes for EEG recording in monkey N. (B) The probability of High tone (80 dB, 1500 Hz) was 20% and that of Low tone (60 dB, 1500 Hz or 80 dB, 1200 Hz) was 80% in block A. The probability of High tone (80 dB, 1500 Hz) was 20% and that of Low tone (60 dB, 1500 Hz or 80 dB, 1200 Hz) was 80% in block B. The order of block A and B was randomized on a day-by-day basis. (C) AEP for 60 dB-tone (blue dashed line) and 80 dB-tone (red dashed line) when interleaved during saline sessions (8016 trials). (D) AEP for 1200 Hz-tone (blue dashed line) and 1500 Hz-tone (red dashed line) when interleaved during saline sessions (8124 trials). (E) Comparison between AEP for 80dB-tone when interleaved with 60dB-tone during saline sessions (red dashed line, 8016 trials) and PCP sessions (red solid line, 3879 trials). Data points with *p*-values (Wilcoxon rank sum test) smaller than 0.01 were indicated by red ticks. (F) Comparison between AEP for 1500Hz–tone when interleaved with 1200 Hz-tone during saline sessions (red dashed line, 8124 trials) and PCP sessions (red solid line, 5630 trials). Same format as in (C). (G) Comparison between AEP for 80 dB-tone when interleaved with 60 dB-tone during saline sessions (green dashed line, 1638 trials) and ketamine sessions (green solid line, 1626 trials). Same format as in (C). (H) Comparison between AEP for 1500 Hz-tone when interleaved with 1200 Hz-tone during saline sessions (green dashed line, 1956 trials) and ketamine sessions (green solid line, 1710 trials). Same format as in (C).

Schizophrenia patients frequently suffer from problems in gating out irrelevant sensory stimuli [30]. To examine the effect of PCP/ketamine on sensory processing in macaques, we recorded EEG signals from the monkeys during tone presentation (Fig. 6A and B). Since the two monkeys were implanted with electrodes in different ways (see Methods), we first examined whether AEP depended on the recording method. We found that whether electrodes were implanted invasively into the skull (monkey N), or simply placed on the surface of the scalp (monkey Y) produced a different signal-to-noise ratio. In particular, AEP from monkey N was much larger and less noisy than AEP from monkey Y (Supplementary Fig. S3E–H), suggesting invasive recordings could significantly improve the signal-to-noise ratio of the collected AEP data.

We next examined whether AEP were correlated with the physical feature of tone (namely, intensity and pitch) under the saline-control condition. We found that larger intensity (80 dB vs. 60 dB) as well as higher pitch (1500 Hz vs. 1200 Hz) significantly evoked stronger peak-to-trough modulation in AEP (Fig. 6C and D). This trend held in both monkeys, although EEG in monkey Y had a relatively weaker signal-to-ratio than in monkey N (Supplementary Fig. S3E–H).

We then examined the drug-injection effect on AEP. For the sound evoking larger AEP (i.e. 80 dB, or 1500 Hz), both drugs significantly increased the modulation gain in monkey N (Fig. 6E–H). Effects were also similar for the sound that evoked weaker AEP (i.e. 60 dB, or 1200 Hz), although the effective size in monkey Y was relatively smaller (Supplementary Fig. S3). Thus, drug applications appeared to amplify AEP to sound stimulus, probably meaning more difficulty in gating out sensory input after PCP or ketamine application.

Our experimental paradigm also allowed to examine the mismatch negativity (MMN), a biomarker widely used in clinics for help identify schizophrenia (Fig. 6B). In particular, sounds with different intensity (60 dB and 80 dB), or pitch (1200 Hz and 1500 Hz) were interleaved within one block, with different probability (see Methods). Briefly, in each block, one of the variables occupied a smaller proportion of trials (20%), serving as the oddball stimulus. Each variable could serve as the oddball stimulus in one block, and serve as the standard, that is, more frequently repeated stimulus in the other block (i.e. 80%). For the same variable (e.g. 80 dB), the difference in AEP between the two blocks was calculated as the MMN index (Supplementary Fig. S4). We found that for sound intensity of 80 dB, the index was significantly reduced by PCP injection for monkey N, particularly for 100–200 ms after tone onset (around the N2 component) (Supplementary Fig. S4A). A similar trend was also found for the ketamine-injection session, yet the effect did not reach statistical significance (Supplementary Fig. S4B). The difference in AEP between 1500 Hz-deviant and 1500 Hz-normal was small (Supplementary Fig. S4C and S4D), thus the MMN curves had too low signal-to-noise ratio and did not show reliable results around the N2 component. Likewise, we failed to find significant change in MMN induced by PCP/ketamine for most conditions in monkey Y, probably due to the weak signal-to-noise ratio signal collected from the noninvasive recordings (Supplementary Fig. S5).

## DISCUSSION

### Oculomotor measurement

Both human and nonhuman primates rely heavily on visual information, and they share very similar visual systems and oculomotor functions that help orient or maintain visual stability in complex environments. In the current study, oculomotor performance in a carefully designed test toolkit was collected from two macaque monkeys before and after PCP/ketamine injections. By analyzing many aspects of the oculomotor behavior in such a complete paradigm including large saccade, smooth pursuit, fixation, microsaccade, and pupil responses, we found robust and similar effects from application of PCP and ketamine, albeit with some difference. In particular, the main change induced by drugs include: (1) reduced anti-saccade accuracy with lagged initiation; (2) decreased saccade speed with increased duration; (3) decreased smooth pursuit gain accompanied by increased saccade occurrence; (4) biased vertical microsaccade toward the lower visual field with increased speed and duration; (5) less stable fixation ability; (6) reduced pupillary response. These various oculomotor abnormalities induced by PCP/ketamine are reminiscent of that observed in schizophrenia patients [11,14,23], suggesting that the drug-induced animal model may share with patients the same dysfunctions in the oculomotor system including cortical, subcortical, and cerebellar areas.

Although the effects of ketamine on some oculomotor behaviors [8,29,31–34] has been tested in monkeys in previous research, to our knowledge here we have the first monkey model on which an extensive parameter range test has been conducted on the same individuals. A thorough and comprehensive analysis of different oculomotor performance on each individual allows to potentially disentangle different types of mental disorders that often share common symptoms. Benson *et al.* and St. Clair *et al.* demonstrated that multiple eye movement measures recorded during smooth pursuit, fixation stability, and free-viewing tasks can differentiate between schizophrenia, depression, bipolar disorder and healthy subjects with approximately 80% predictive accuracy [35]. When distinguishing only between schizophrenia and healthy subjects, the accuracy can reach up to 98% [13]. In our current animal model study, we show that drug-administration state (saline, PCP, ketamine) could be reliably separated for both of the animals in a high dimensional space of many oculomotor parameters and a PCA-based multi-class SVM method can achieve a high level of accuracy of 93.4% in identifying the three drug-injection states. Thus, it may potentially help simulate other mental disorders by examining their patterns in a high dimensional space, which may lead to disentanglement. Furthermore, we show that training a decoder using one animal's data could generate a high performance when testing the other animal's data, although the two animals exhibit quite a difference in their baseline oculomotor behavior. Thus, our model allows data to transfer across subjects typically suffering from inter-group variability, providing potential values for future clinical applications. Overall, macaques share many analogous functions including oculomotor behavior, visual system, and cognitive functions with humans. Constructing a monkey model with a high dimension of oculomotor parameters could provide much convenience for future application of pharmacological and physical therapy tests.

The oculomotor test toolkit in our current study contains several tasks that involve both cognitive and motor functions. The widespread abnormalities in oculomotor performance induced by drug injections thus reflect deficits in the underlying structures involving multiple levels of brain regions. For example, both anti-saccade and delayed-saccade tasks demand cognitive control. The increased error rate and longer saccade latency thus reflect deficits in cognitive control functions of attention and inhibition, which may be mediated by the dorsolateral prefrontal cortex (dlPFC [36]). The slower saccade velocity with longer saccade duration indicates that the deficit may be mediated by frontal eye fields (FEFs [37]) or cerebellum, which controls the temporal property of saccades [38]. For smooth pursuit, coordination of multiple cognitive processes is required for being able to track moving objects smoothly and accurately by matching target velocity with the movement of the eyes. Unlike the saccade, pursuit involves the extrastriate visual cortex of the middle temporal area (MT) and the medial superior temporal area (MST) that are responsible for processing visual motion information [39]. FEF and dlPFC are also required to control the pursuit gain and to predict target movement. Thus, the reduced pursuit gain in our study indicates deficits in these pursuit-related areas. For microsaccades during fixation, they share many common neural bases with regular saccades, yet microsaccades are involuntary [25]. The spatial and temporal abnormalities of microsaccades during fixation may reflect alterations in spontaneous neural activity of the cortical-subcortical-cerebellar circuitry. Interestingly, we found that PCP/ketamine induced a bias of the microsaccade direction toward the lower visual field, a phenomenon that is also observed in schizophrenia patients [14]. Finally, pupillary response is a reliable oculomotor index of cognitive load, involving the Locus Coeruleus-Norepinephrine (LC-NA) system that is responsible for releasing neurotransmitter NE and plays a crucial role in regulating cognitive functions [12]. Reduced pupillary response has been observed in patients with schizophrenia that is thought to be related with negative symptoms [23]. Here we also observed reduced pupil size with shrinking pupillary response, suggesting dysfunction in the LC-NA system induced by PCP or ketamine.

Although PCP and ketamine produced similar results in many aspects in the current study, we also noticed some difference between the two drugs. For example, ketamine injection did not significantly reduce the correct rates in pro-saccade and delayed saccade tasks, while PCP exhibited a notable decrease in accuracy (Fig. 1). The reasons for causing different effects might be variations in the dosage of the two drugs and differences in their binding sites with receptors, and ketamine's relatively shorter half-life compared to PCP [40]. The effects of ketamine typically peak around 10 minutes after injection and most disappear within 1 to 2 hours ([31], also see Supplementary Fig. S7). All these may allow PCA to effectively differentiate oculomotor effects between the two drugs [5]. Interestingly, in recent years ketamine received much attention because it exhibits a unique, immediate antidepressant effect, which appears ∼30 minutes after injection and could sustain for as long as 7 days. The difference in onset times for ketamine-induced schizophrenia-like symptoms and particularly its long-lasting antidepressant effects indicates that the two types of effects may have distinct neural pathways [40]. However, to date it is still inconclusive about whether the antidepressant effect is dependent on dissociative effects, which requires further investigation [41]. Finally, we have applied racemic ketamine used in the current study, which is composed of both S-ketamine and R-ketamine. S-ketamine, rather than R-ketamine, induces dopamine release from the neuronal terminals in the striatum [42] and interacts significantly with μ-opioid receptors (MORs) [43], which may contribute to the stronger psychotomimetic effects of racemic ketamine. Future studies could explore the dependence of oculomotor performance on drug dose, as well as the effects of R-ketamine and S-ketamine on eye movements.

### EEG measurement

Deficits in sensory/sensorimotor gating in schizophrenia patients have been frequently reported in previous research [30,44]. Two widely used biomarkers—P50 in ERP and pre-pulse inhibition (PPI) in EMG—can test the integrity of sensory/sensorimotor gating abilities. For example, P50 was remarkably reduced in normal controls when the same sound was immediately replayed due to adaptation, and this reduction was smaller in schizophrenia patients. However, P50 is usually small in its magnitude and thus its reliability easily suffers from a low signal-to-noise ratio problem [45]. Thus, we tested whether it is possible to detect signals in other EEG components on our NHP model. We found that our stimuli evoked reliable AEP response not only in P50, but also in other components, such as N100 and P200. Importantly, we observed robust enhancements in AEP under PCP/ketamine injection conditions, reminiscent of the relieved P50 reduction in response to the replayed sound in patients.

For PPI, usually a loud sound (e.g. 115 dB) is used to evoke the startling response, for example, jumping up in the rodent study [44,46]. For normal controls, the startling response could be reduced if it occurs right after (30–120 ms) a neutral pre-pulse that typically does not evoke startling responses, an effect known as PPI. In contrast, the PPI effect is much weaker in schizophrenia patients [46]. PCP application in monkeys also led to similar reduction in PPI [47]. We did not measure the PPI in our current study, yet the increased AEP after PCP/ketamine injection is consistent with the PPI effects reported in the previous studies on schizophrenia patients and on PCP-induced monkeys.

Compared with P50 and PPI, MMN could be conceptualized as an index to gate in meaningful information in the environment. Even though MMN has been reliably established in humans, its counterpart in nonhuman animals so far has been controversial [48,49]. For example, careful examination of multi-unit activity in the primary auditory cortex suggested that the increased responses to the infrequent tone was related more to adaptation than to novelty detection [49]. Even though MMN was reduced after PCP administration in our result as previously reported by local infusion of PCP into the cortex [48], we cannot rule out the contribution from stimulus-specific adaptation [50]. More experiments, such as the many-standard control and the cascade control, may have to be conducted to tease out the true MMN component [50]. Furthermore, because AEP itself depended on the recording method, sound intensity and pitch, the difference in AEP (i.e. MMN) became more vulnerable when the SNR was low and thus less robust than AEP. Therefore, we concluded that measuring changes in AEP offers a window to monitor sensory gating-out capacity and be more robust than MMN, thus beingh a good biomarker for schizophrenia.

## MATERIALS AND METHODS

For detailed materials and methods, please see the Supplementary data.

## Supplementary Material

nwad255_Supplemental_File
