# Supplementary material for: Decoding effects of psychoactive drugs in a high-dimensional space of eye movements in monkeys
Source: Natl Sci Rev. 2023 Oct 3;10(11):nwad255. doi: 10.1093/nsr/nwad255 (PMC10689211; doi:10.1093/nsr/nwad255)
Supplement: nwad255_Supplemental_File [file nwad255_supplemental_file.docx]

Supplementary data

# ***Methods***

## Subjects

Experiments were performed on two male rhesus monkeys (Macaca mulatta), weighted 9–12 kg. The monkeys were chronically implanted with a circular molded lightweight plastic ring above cranium for head fixation. After recovery, the animals were trained to seat in a custom-built primate chair with their heads restrained and performed the behavioral tasks. All animal procedures were approved by the Animal Care Committee of Institute of Neuroscience, Chinese Academy of Sciences (#CEBSIT-2022010).

## Apparatus

During behavior training, monkeys were comfortably seated in a chair, facing a LCD visual display (SAMSUNG DB55E, 75 Hz, 1920 × 1080) in front of them with a viewing distance of 65 cm. The visual display was 120 cm wide and 68 cm high, occupying a visual angle of approximately 85° in width and 55° in height. Visual stimulus was produced through MonkeyLogic (NIMH).

## Experimental paradigm

### Oculomotor tasks

Monkeys typically were instructed to perform 5 oculomotor tasks each day. The tasks were presented in the following sequence: delayed saccade, anti- and pro-saccade, linear pursuit, Lissajous pursuit, and free viewing tasks. The five tasks were illustrated in the following:

***Delayed saccade task****:* Monkeys were instructed to fixate on a cyan circular central fixation point with a diameter of 0.5° for 500 msec. Subsequently, a square cue with same color randomly presented in one of the four positions (left, right, up or down) with eccentricity of 10°. After the peripheral target appeared, the monkeys should still maintain central fixation for an additional 1500 ms, until the fixation point disappears, indicating a go signal. After that, the monkeys should make a saccade to the peripheral target.

***Anti- and pro-saccade tasks****:* Each trial started with the monkeys fixating a colored (red or blue) circular target with a diameter of 0.5° placed at the center of the visual display. The monkey needed to fixate the central target for 500 ms within an electronic window of 2° in diameter. Any blinks or eye movements that exceeded the window would lead to failed performance, and the current trial would be terminated. After the 500 ms of fixation, the central fixation point jumped to either left or right with eccentricity of 10°. In anti-saccade trials, the fixation and the peripheral targets were red, indicating that monkeys needed to make a saccade in the opposite direction of the peripheral target. In pro-saccade trials, the central and peripheral targets were cued as blue, indicating that the monkeys needed to saccade to the peripheral target. The anti- and pro-saccade trials were interleaved within one experimental session with a ratio of 2 to 1. There were totally 240 trials in each experimental session.

***Linear smooth pursuit****:* Monkeys were instructed to fixate their gaze on a green spot with a diameter of 0.5°. The target was presented with equal probability in one of the four positions (up, down, left, or right) with eccentricity of 10°. After fixation of 500 ms, the target moved towards the center of the visual display along a straight line, with a constant speed of 10°/s. The trial lasted 2 seconds, thus, the target would move across the center of the visual display and continuously moved to the other side. Monkeys were required to track the moving target within an electronic window of 5° in diameter. There were 60 trials in one experimental session.

***Lissajous smooth pursuit****:* Monkeys were required to track a target that was continuously moving, similar to that in the linear pursuit task. Yet the target was moving in two dimensions along a Lissajous curve (see **Figure 2D**, dot line), which was a periodic motion trajectory based on two sinusoidal curves. In particular, the relationship between the horizontal and vertical position of target and time was as following:

$$x\left( t \right)=3\sin\left( \pi t \right)$$

$y\left( t \right)=-3cos(2\pi t + \pi/2)$

, where $x\left( t \right)$ and $y\left( t \right)$represented the horizontal and vertical positions at time *t*, respectively. Thus, unlike the linear pursuit with a constant speed, the velocity in the Lissajous task actually kept changing across the stimulus duration. The target lasted moving for 2 seconds in each trial, and the trial would be terminated if the monkeys exceeded an electronic window of 5° during pursuit. There were 30 trials in one experimental session.

***Free viewing*** ***task****:* In all aforementioned tasks, the animals were instructed to make specific eye movements. In the free viewing session, the monkeys were largely allowed to freely move their eyes when presented with a picture (8°×8° in width and height) at the center of the display. We used six classical illusory images, such as rotating snakes and Hermann grid, which tend to evoke illusory motion perception [1-4], and six non-illusory images. All these images have now been uploaded to the supplementary materials (**Figure S3A**). Each image was presented 10 times by lasting 2.5 second. Although the animals had large freedom in their oculomotor behavior, to encourage viewing, we still put a limit by instructing the animals to avoid blinks, or too large eccentric gaze beyond 10° away from the center of the picture.

### *EEG measurement and Sound stimuli*

In the EEG experiment, we used a passive auditory stimulation paradigm by presenting tones with different frequency (low: 1200 Hz; high: 1500 Hz) or different intensity (low: 60 dB; high: 80 dB) to monkeys in a sound-isolated, dimly lit room. The sound stimulus lasted 100 ms period in each trial. The sound paradigm contained two experiments. In the intensity experiment, there were two blocks. One block contained 80% trials with low intensity tone (60 dB), while the rest 20% trials had high intensity tone (80 dB). The other block has the reversed probability of the low and high intensity tones. The two blocks were randomly ordered across days. The frequency was always 1500 Hz. In the other frequency experiment, the basic paradigm was similar to the intensity experiment, except that the intensity was always fixed at 80 dB, while the frequency was varied between 1200 Hz and 1500 Hz. Stimulus presentation was controlled by NIMH MonkeyLogic using a Dell Workstation. Tones were presented using a built-in speaker in the LCD monitor (Samsung DB55E). During sound presentation, the monkeys were head-fixed and maintained central fixation. The fixation target was a white circle (0.5° in diameter) on a black background in the visual display.

EEG signals were recorded using Medusa (Bio-Signal Technologies) EEG/EMG acquisition device with the Athena software (Bio-Signal Technologies). For monkey N, three titanium screws were implanted along the mid-sagittal plane on the skull, and three Ag/AgCl electrodes were applied in the posterior regions as reference and ground electrodes (Figure. 6A). For monkey Y, six Ag/AgCl electrodes were placed around the head for EEG recording. The monkeys sit comfortably in the primate chair with head fixated and faced toward the visual display. EEG signals went through a bandpass filter (low bound: 0.1 Hz; high bound: 30Hz) and a notch filter (50Hz) during data acquisition. The raw data were then converted to EDF format for later analysis. Since the three titanium screws were inserted into the skull of monkey N, we found that the signals were much clean in these channels compared to that in monkey Y with electrodes simply attached to the surface of the skull. Thus, we focused our analysis on monkey N. In particular, EEG data were analyzed using the fieldtrip toolbox and customized code in Matlab. The analysis procedure included preprocessing (rereferencing, band-pass filtering, segmentation, etc.) before calculating ERPs for each condition. To balance the number of trials for different conditions and to minimize the fluctuation in the signal, we included all artifact-free trials for the tone with a lower probability (20%). For the tone with a higher probability (80%), only the trials leading by the less frequent tone were included.

## *PCP and Ketamine injection*

PCP (The third Research Institute of Mimnistry of Public Security, Shanghai) was diluted with saline to reach concentration of 3 mg/ml, and was delivered to the animals through lower limb muscle injection. The total amount of drug was applied to each monkey with a dose of 0.3 mg/kg. This dose was found to produce significant drug effects and commonly used as the injection dosage for acute PCP studies in monkeys [5-7]. Immediately after PCP injection, we found that monkeys refused to play any oculomotor tasks in the system, so we started behavioral tasks one hour after PCP injection (**Figure S1 A**).

Ketamine was diluted with saline to reach concentration of 8 mg/ml, and was delivered to the animals with a dose of 0.8 mg/kg. The other procedure was similar to the PCP (**Figure S1 A**). As a control, saline was also applied with the same procedure with a dose of 0.1 ml/kg. The injection sequences for the PCP and ketamine trials during oculomotor tasks are shown in **Figure S1 B**.

For EEG recordings, they were performed on separate days after all the oculormotor experiments were done.

## Data analysis

### *Saccade and microsaccade detection*

Eye position data were collected by infrared eye trackers (Eyelink, Canada) with a temporal resolution of 1K Hz for the sampling freqency. The data were filtered using a zero-phase two-pole Butterworth low-pass filter with a cutoff of 40 Hz. Based on these data, eye movement velocity and acceleration signals were then calculated as the first and second derivative over time. Notably, saccade not only occurred in saccade-related tasks, but can also happened in smooth pursuit tasks. Thus, we detected these saccade behaviors in all tasks with velocity and acceleration thresholds [8]. In particular, a saccade was defined when the velocity was beyond 30°/sec and the acceleration was beyond 800°/sec^2^, and this behavior lasted continuously for at least 10 msec.

Other than the regular saccade, the microsaccade typically refers to those small saccade made during central fixation that cannot be successfully suppressed although they are within the required fixation window. To characterize these fixational eye movements, we used velocity-based algorithm [9] where an elliptic threshold was determined in 2D velocity space based on the horizontal and vertical velocities of eye movement. In particular, threshold ($\eta_{x,y}$) for horizontal and vertical velocity was calculated by:

$\eta_{x,y}= {\lambda\cdot\sigma}_{x,y}$

$\sigma_{x,y}^{2}=\left\langle{(v_{x,y}-\left\langle v_{x,y} \right\rangle)}^{2} \right\rangle$

, where$v_{x,y}$ represents the horizontal and vertical velocities of eye movement, and $\left\langle. \right\rangle$denotes the median estimator. Here λ was set to be 5. Similar to the regular macrosaccade, a microsaccade was identified only when 10 or more data samples were continuously outside the ellipse defined by the horizontal and vertical threshold.

During the free viewing session, monkeys’ eye movements can be characterized by fixations, microsaccades (i.e., fixational saccades), and regular macrosaccades (i.e., exploratory saccades). These saccades were detected with the same algorithms as described above.

### *Quantify oculomotor performance*

*Saccade task:* Up to five parameters including accuracy of saccade direction, reaction time of the initiated saccade, averaged saccade velocity, saccade duration and saccade amplitude were calculated. In particular, reaction time was defined as the time point when a saccade was initiated after the central fixation point was disappeared.

*Pursuit task:* Two parameters of saccade frequency and pursuit gain were calculated. Each saccade was detected according to algorithms as described in the previous section. For pursuit gain, it was defined as the ratio of the averaged pursuit velocity to the target velocity in the linear pursuit task. Saccades were excluded. In Lissajous pursuit task, however, since the target velocity is composed of two different sinusoidal functions in the horizontal and vertical directions, the gain was calculated separately in the two directions. In particular, we fit sinusoidal functions to the horizontal and vertical components of the eye tracking velocity. Then each gain was defined as the ratio of the fitted peak to the target velocity [10].

*Central fixation:* We analyzed three oculomotor behaviors including microsaccade, fixation stability, and pupillay response. For microsaccade, four parameters of direction, averaged velocity, duration and amplitude were calculated.

For fixation stability, it was quantified by the bivariate contour ellipse area (BCEA) of the overall eye positions during central fixation. In particular, BCEA is defined as:

$$BCEA =\pi\chi^{2}\sigma_{x}\sigma_{y}{(1-\rho^{2})}^{1/2}$$

, where $\sigma_{x}$ and $\sigma_{y}$ are the standard deviations of the eye position in the horizontal (x) and vertical (y) directions respectively. $\rho$ is the Pearson product-moment correlation of two position components, and $\chi^{2}=2.291$is the Chi-squared value corresponding to a probability value of $p=0.682$ (±1 standard deviation). The BCEA represents the area that encompasses 68.2% of the eye positions. A log10 transformation was used to normalize the resulting BCEAs. A larger BCEA represents a less stable fixation.

For pupillary response, raw pupil size data were collected by infrared eye tracker for each monkey. A baseline was defined as the average pupil size of the first 200 ms of central fixation. Pupillary response was then defined as the pupil-size change relative to the baseline.

*Free viewing:* We plotted the averaged eye position distribution in two-dimensional space, and analyzed the occurrence frequency and angle of saccadic eye movements.

## Principal component analysis and classification

We used machine learning method to classify the three injection states of saline, PCP and ketamine injection based on a dataset including a total of 333 samples from the two monkeys. The oculomotor parameters measured from the oculomotor tasks (see **Figure 5C**) were used as the inputs of principal component analysis (PCA).

After PCA, we employed one-way analysis of variance (ANOVA) to analyze the principal components (PCs) and obtain the statistical differences among the PCs in three injection states. To eliminate redundant features and prevent overfitting, the three principal components with the smallest p-values were choose as the input for a multi-class Gaussian support vector machine (SVM) decoder for classification. Because a principal component (PC) was the linear combination of the oculomotor parameters and the corresponding PC coefficients, the weights of oculomotor parameters loaded onto the these three PCs can be defined as$w_{j}= \frac{\sum_{i}^{7} \left| L_{ij} \right|C_{i}}{\sum_{i}^{7} C_{i}}$, where $w_{j}$ is the weight of the $j^{th}$ oculomotor parameter, $C_{i}$ is the contribution rate of the $i^{th}$ PC, $L_{ij}$ is the ${ij}^{th}$ PC coefficient, and $\left| \cdot\right|$ represents the absolute value. Leave-one-out cross validation (LOOCV) was conducted to estimate the performance of the classifiers.

Furthermore, to test the transferability of the model, we performed additional analysis by training data from one monkey and testing drug-injection state in the other monkey. The classification was performed using Statistics and Machine Learning Toolbox in MATLAB 2020a.

## Reference

1. Spillmann, L., *The Ouchi-Spillmann illusion revisited.* Perception, 2013. **42**(4): p. 413-29.

2. Faubert, J. and A.M. Herbert, *The peripheral drift illusion: a motion illusion in the visual periphery.* Perception, 1999. **28**(5): p. 617-21.

3. Spillmann, L., *The Hermann grid illusion: a tool for studying human perspective field organization.* Perception, 1994. **23**(6): p. 691-708.

4. Gori, S., et al., *A new motion illusion based on competition between two kinds of motion processing units: the accordion grating.* Neural Netw, 2011. **24**(10): p. 1082-92.

5. Frederick, D.L., et al., *Acute behavioral effects of phencyclidine on rhesus monkey performance in an operant test battery.* Pharmacol Biochem Behav, 1995. **52**(4): p. 789-97.

6. Jentsch, J.D., et al., *Phencyclidine increases forebrain monoamine metabolism in rats and monkeys: modulation by the isomers of HA966.* J Neurosci, 1997. **17**(5): p. 1769-75.

7. Zick, J.L., et al., *Blocking NMDAR Disrupts Spike Timing and Decouples Monkey Prefrontal Circuits: Implications for Activity-Dependent Disconnection in Schizophrenia.* Neuron, 2018. **98**(6): p. 1243-1255 e5.

8. Krauzlis, R.J. and F.A. Miles, *Release of fixation for pursuit and saccades in humans: evidence for shared inputs acting on different neural substrates.* J Neurophysiol, 1996. **76**(5): p. 2822-33.

9. Engbert, R. and K. Mergenthaler, *Microsaccades are triggered by low retinal image slip.* Proc Natl Acad Sci U S A, 2006. **103**(18): p. 7192-7.

10. Lencer, R. and P. Trillenberg, *Neurophysiology and neuroanatomy of smooth pursuit in humans.* Brain Cogn, 2008. **68**(3): p. 219-28.

# Supplementary Figures


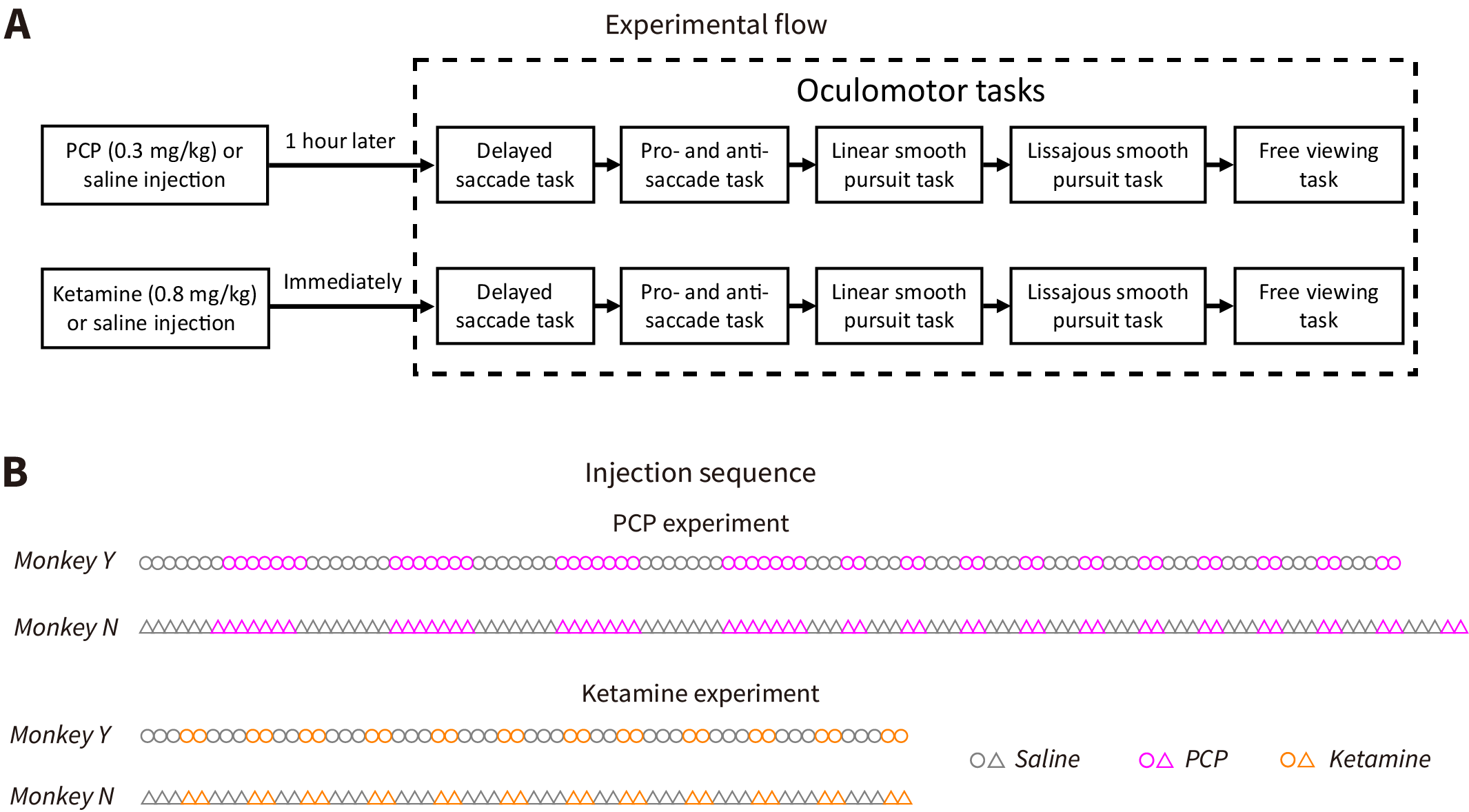


**Figure S1**. The flowchart of drug injection and oculomotor task. **(A)** The PCP experiment and the ketamine experiment are conducted separately. The ketamine trials started after all PCP trials were completed. Daily drug injections were conducted, starting with several consecutive days of saline injections, followed by several days of daily PCP or ketamine injections. To eliminate any interference from drug carryover effects on the saline control experiments, the monkeys were allowed to rest for 2 days without any injection before starting the saline trials. After drug or saline injection, monkeys were instructed to perform 5 oculomotor tasks presented in the following sequence: delayed saccade, anti- and pro-saccade, linear pursuit, Lissajous pursuit, and free viewing tasks. Because monkeys refused to perform any oculomotor tasks immediately after PCP injection, the oculomotor tasks were started one hour after PCP or saline injection in the PCP experiment. **(B)** The injection sequences for the PCP and ketamine experiments. In the PCP experiment, monkey Y received a total of 106 injections, with 48 injections of PCP and 58 injections of saline. Monkey N received a total of 111 injections, with 50 injections of PCP and 61 injections of saline. In the ketamine experiment, both monkey Y and monkey N received a total of 58 injections, with 24 injections of ketamine and 34 injections of saline.


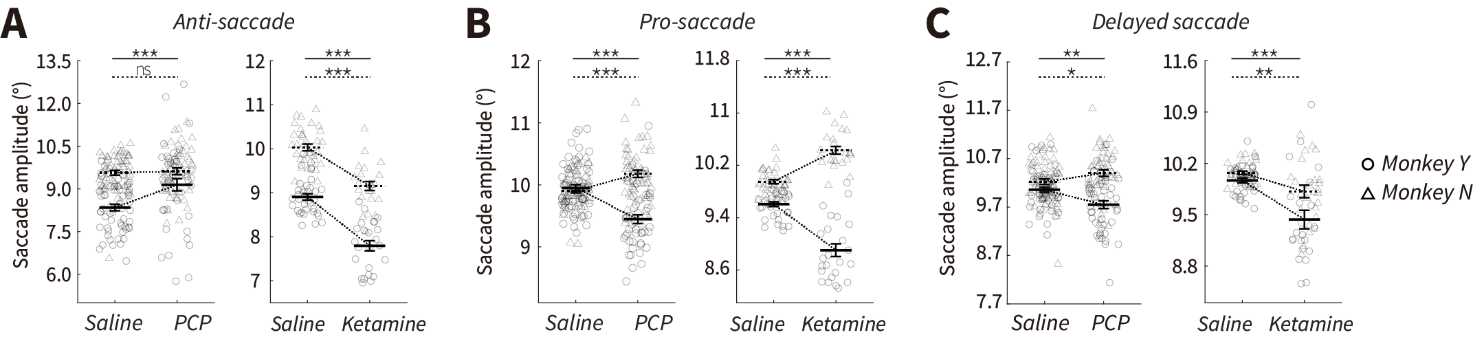


**Figure S2**. PCP and ketamine effects on saccade amplitude. **(A)** PCP (left) and ketamine (right) exhibited different effects on saccade amplitude in the anti-saccade task. **(B)** Monkeys showed different responses to PCP (left) and ketamine (right) in the pro-saccade task that PCP and ketamine decreased the saccade amplitude of monkey Y, but increased the saccade amplitude of monkey N. **(C)** In the delayed saccade task, the saccade amplitude was decreased in monkey Y and increased in monkey N after PCP injection (left), while ketamine decreased the saccade amplitudes of both monkeys (right). Circle and triangle represent data from two monkeys. Solid, dark symbol represent mean value and the open, light symbols are data from each session. Error bars indicate ± SEM. Statistical significance: *0.01<p<0.05; **0.001<p<0.01; ***<0.001; ns, p>0.05 (solid line for monkey Y; dotted line for monkey N).

**
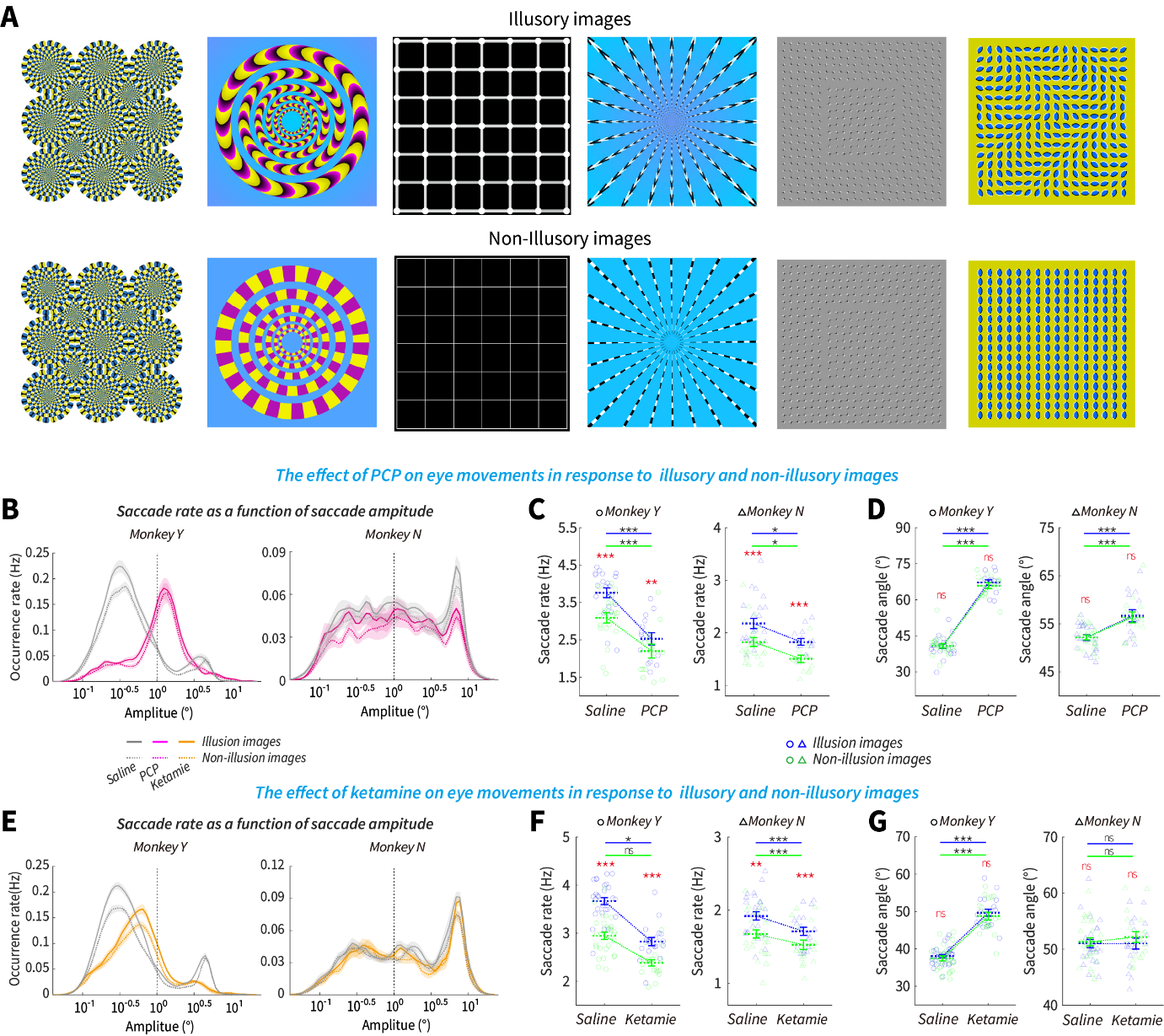
 Figure S3**：Eye movements of monkeys in response to illusory and non-illusory images. **(A)** Illusory and non-illusory images**. (B)** Proportion of saccade amplitude before (grey curves) and after PCP injection (color curves). Solid and dashed lines represent responses to illusory and non-illusory images, respectively. **(C-D)** The overall saccade rate **(C)** and direction away from horizontal meridian (saccade angle) **(D)** before and after PCP injection. Blue and green markers represent illusory and non-illusory images, respectively. The red * indicates the statistical significance between the saccade parameters to illusory and non-illusory images. The black * indicates the statistical significance between the saccade parameters before and after PCP injection. (**E-G**) same as **(B-D)** but for saline and ketamine results. Circle and triangle represent data from two monkeys. Solid, dark symbol represent mean value and the open, light symbols are data from each session. Error bars indicate ± SEM. Statistical significance: *0.01<p<0.05; **0.001<p<0.01; ***<0.001; ns, p>0.05.


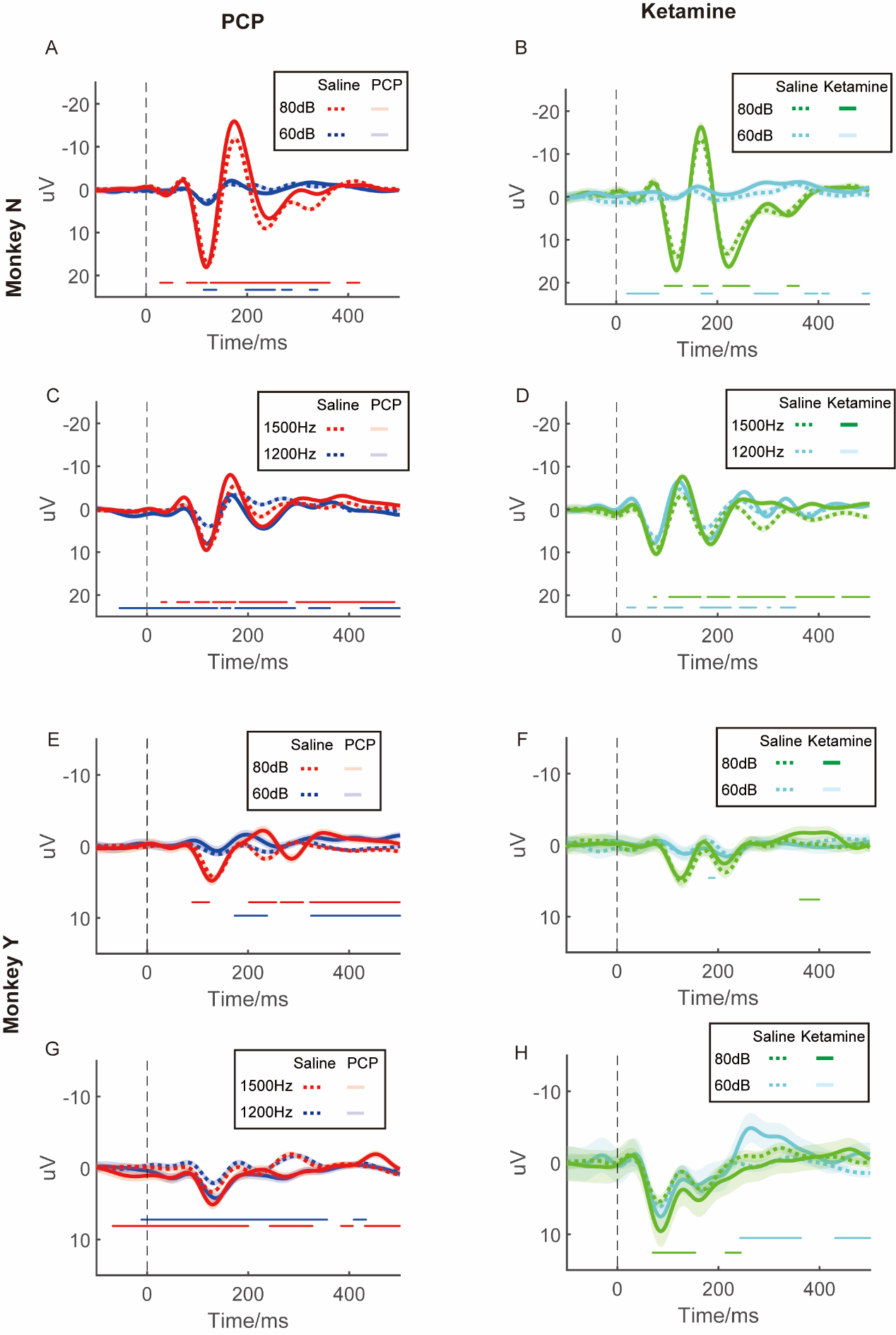


**Figure S4**. The effect of PCP/ketamine administration on AEP in monkey N and monkey Y.

1. AEP for 60dB-tone (blue lines) and 80dB-tone (red lines) when interleaved during saline sessions (dashed, 8016 trials) and PCP sessions (solid, 3879 trials) for monkey N.
2. AEP for 60dB-tone (cyan lines) and 80dB-tone (green lines) when interleaved during saline sessions (dashed, 1638 trials) and ketamine sessions (solid, 1626 trials) for monkey N.
3. AEP for 1200Hz-tone (blue lines) and 1500Hz-tone (red lines) when interleaved during saline sessions (dashed, 8124 trials) and PCP sessions (solid, 5630 trials) for monkey N.
4. AEP for 1200Hz-tone (cyan lines) and 1500Hz-tone (green lines) when interleaved during saline sessions (dashed, 1956 trials) and ketamine sessions (solid, 1710 trials) for monkey N.
5. AEP for 60dB-tone (blue lines) and 80dB-tone (red lines) when interleaved during saline sessions (dashed, 7550 trials) and PCP sessions (solid, 3450 trials) for monkey Y.
6. AEP for 60dB-tone (cyan lines) and 80dB-tone (green lines) when interleaved during saline sessions (dashed, 1568 trials) and ketamine sessions (solid, 1372 trials) for monkey Y.
7. AEP for 1200Hz-tone (blue lines) and 1500Hz-tone (red lines) when interleaved during saline sessions (dashed, 5432 trials) and PCP sessions (solid, 3488 trials) for monkey Y.
8. AEP for 1200Hz-tone (cyan lines) and 1500Hz-tone (green lines) when interleaved during saline sessions (dashed, 2072 trials) and ketamine sessions (solid, 530 trials) for monkey Y.


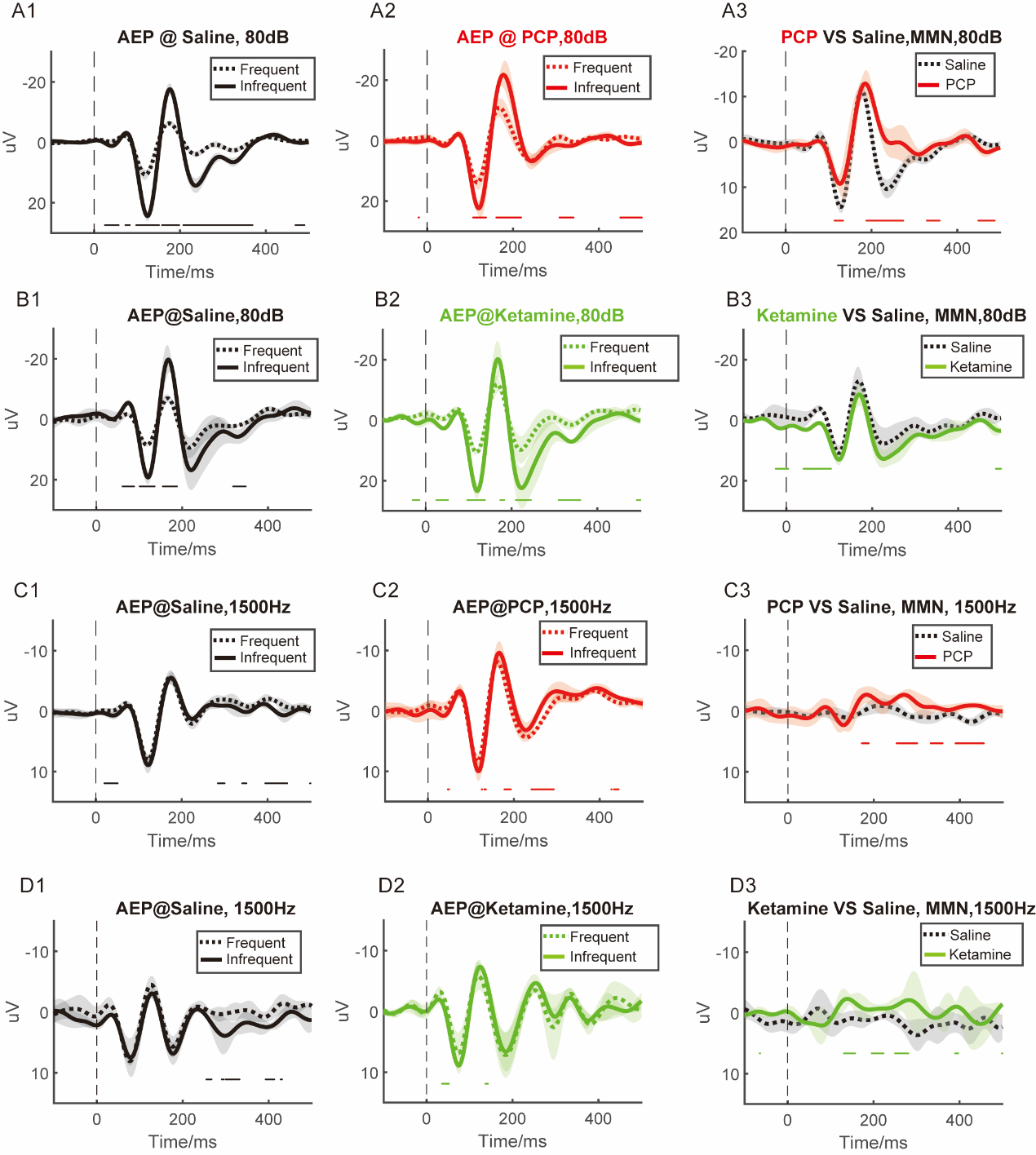


**Figure S5**. The effect of PCP/ketamine administration on MMN in monkey N.

1. The effect of PCP on MMN when 80dB tone was interleaved with 60dB tone.

(A1) The comparison of AEP for 80dB tone when served as the frequent stimulus (solid black line) versus the infrequent stimulus (dashed black line) under the saline condition (18 sessions). Data points with p values (Wilcoxon rank sum test) smaller than 0.05 were indicated by horizontal ticks. The difference between the two curves is the MMN curve.

(A2) The comparison of AEP for 80dB tone when served as the frequent stimulus (solid black line) versus the infrequent stimulus (dashed black line, 18 sessions) under the PCP condition (8 sessions). Data points with p values (Wilcoxon rank sum test) smaller than 0.05 were indicated by horizontal ticks.

(A3) The comparison of MMN under the saline condition (dashed black line) and the PCP condition (solid red line). Data points with p values (Wilcoxon rank sum test) smaller than 0.05 were indicated by horizontal ticks.

1. The effect of ketamine on the MMN when 80dB tone was interleaved with 60dB tone. Same format as in A. n(ketamine) = 4 sessions; n(Saline) = 4 sessions.
2. The effect of PCP on the MMN when 1500Hz tone was interleaved with 1200Hz tone. Same format as in A. n(PCP) = 12 sessions; n(Saline) = 18 sessions.
3. The effect of ketamine on the MMN when 1500Hz tone was interleaved with 1200Hz tone. Same format as in A. n(ketamine) = 4 sessions; n(Saline) = 6 sessions.


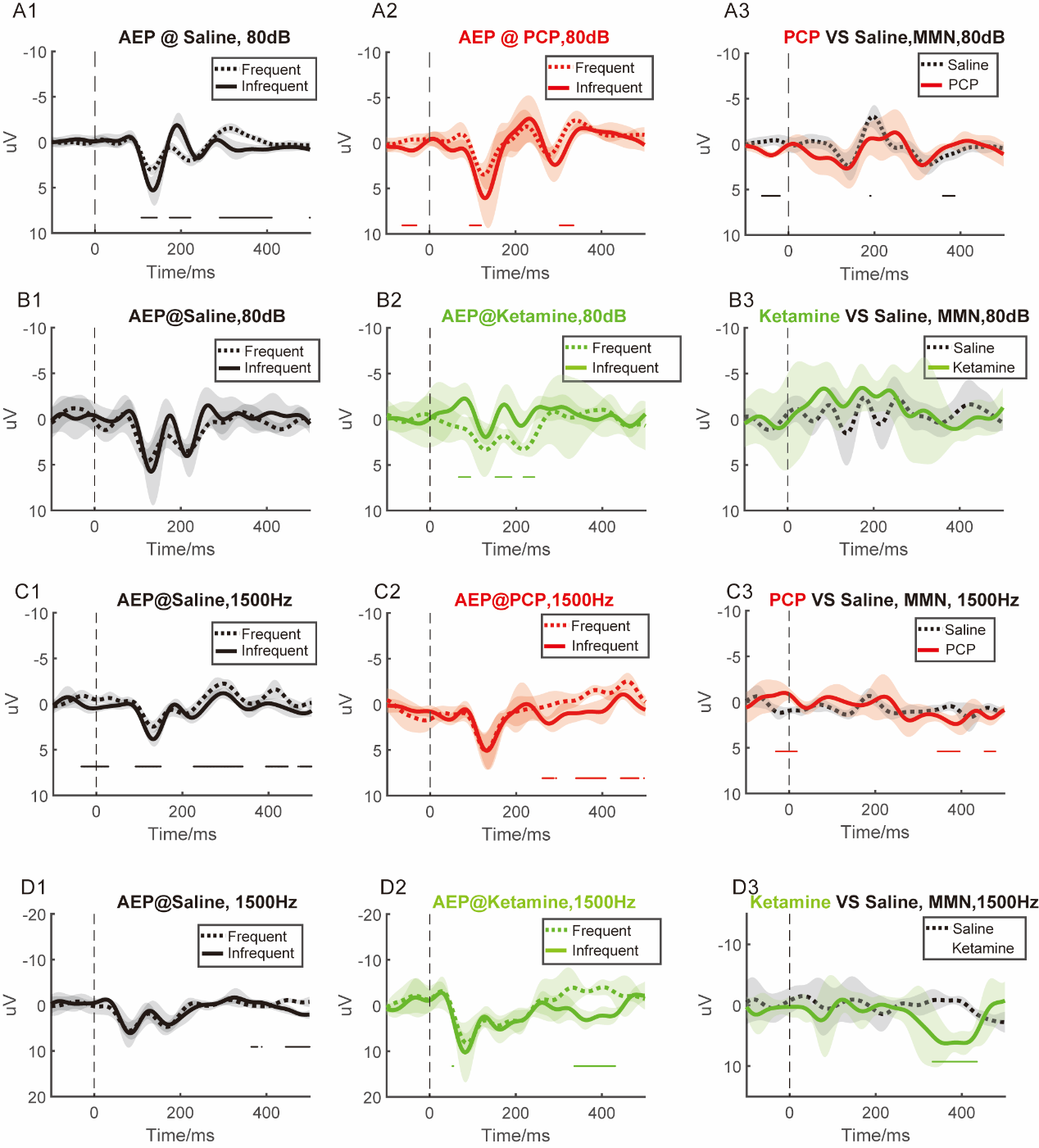


**Figure S6**. The effect of PCP/ketamine administration on MMN in monkey Y.

1. The effect of ketamine on the MMN when 80dB tone was interleaved with 60dB tone. Same format as in Figure S2A. n(PCP) = 8 sessions; n(Saline) = 18 sessions.
2. The effect of ketamine on the MMN when 80dB tone was interleaved with 60dB tone. Same format as in Figure S2A. n(ketamine) = 4 sessions; n(Saline) = 4 sessions.
3. The effect of PCP on the MMN when 1500Hz tone was interleaved with 1200Hz tone. Same format as in Figure S2A. n(PCP) = 10 sessions; n(Saline) = 22 sessions.
4. The effect of ketamine on the MMN when 1500Hz tone was interleaved with 1200Hz tone. Same format as in Figure S2A. n(ketamine) = 4 sessions; n(Saline) = 6 sessions.


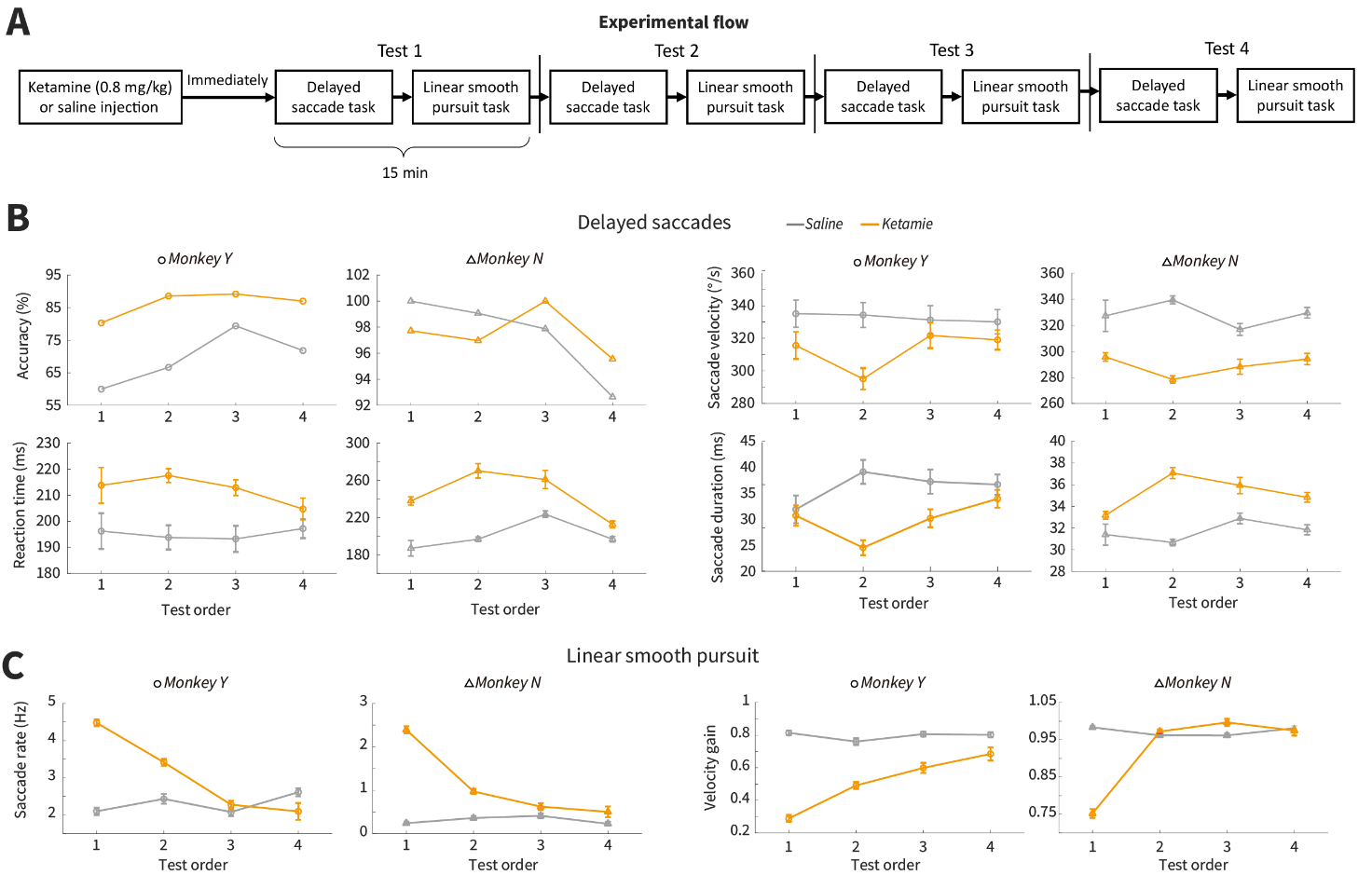


**Figure S7:** Timecorse of ketamine effects on delayed saccade and linear smooth pursuit. **(A)** Flow-chart of the experiment. The delayed saccade task and linear smooth pursuit task took approximately 8 minutes and 7 minutes, respectively. Each task was completed in 4 sets. **(B)** The effect of ketamine on reaction time, saccade velocity and saccade duration reached its peak during the second set of tests and then diminished in the subsequent third and fourth sets of tests (orange represents ketamine, gray represent ketamine)**. (C)** The effects of ketamine on linear pursuit, including an increase in saccade rate and a reduction in pursuit gain, are most pronounced in first set of tests, occurring around 8-15 minutes after ketamine injection.
